# Overview of molecular signatures of senescence and associated resources: pros and cons

**DOI:** 10.1002/2211-5463.70134

**Published:** 2025-10-04

**Authors:** Orestis A. Ntintas, Sylvia Vagena, Pavlos Pantelis, Giorgos Theocharous, Russel Petty, Konstantinos Evangelou, Vassilis G. Gorgoulis

**Affiliations:** ^1^ Molecular Carcinogenesis Group, Department of Histology and Embryology, Medical School National and Kapodistrian University of Athens Athens Greece; ^2^ Intelligencia Inc New York NY USA; ^3^ Vascular Unit, First Propedeutic Department of Surgery National and Kapodistrian University of Athens, Hippocration Hospital Athens Greece; ^4^ Ninewells Hospital and Medical School, University of Dundee Dundee UK; ^5^ Biomedical Research Foundation, Academy of Athens Athens Greece; ^6^ Faculty Institute for Cancer Sciences, Manchester Academic Health Sciences Centre University of Manchester Manchester UK

**Keywords:** biomarkers, computational models, senescence, senescence molecular signatures

## Abstract

The accurate detection of cellular senescence is of paramount importance given its involvement in aging and age‐related pathologies. Over the years, a variety of markers and methodologies have been developed to address this issue. Initially, wet‐lab assays, dealing with single morphological traits and molecular markers, were implemented, though exhibiting technical challenges and ineffectiveness in identifying the inherently complex senescence phenotype. Recent developments led to the adoption of combinatorial approaches in the form of multimarker guideline algorithms, effectively bypassing these obstacles. Moreover, technological advances have facilitated the emergence of molecular signatures that exploit the large amount of data generated in the last decades to increase our awareness of this phenomenon and its consequences. Due to the overwhelming expansion of these signatures, we performed an analysis of their advantages and disadvantages, and here, we discuss future improvements.

## Graphical abstract

Cells can enter a stress response state termed cellular senescence that is involved in various diseases and aging. Detecting these cells is challenging due to the lack of universal biomarkers. This review presents the current state of senescence identification, from biomarkers to molecular signatures, compares tools and approaches, and highlights necessary steps to develop a universal senescence detecting method.

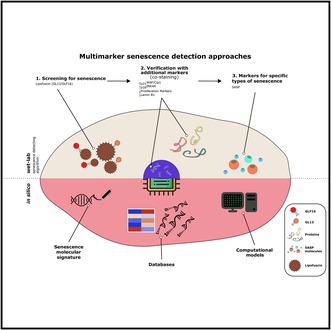

**Abbreviations**
4‐HNE: 4‐hydroxynonenal
ALISE: accumulation of lipids in senescence
ARF: alternate reading frame protein
ATM: ataxia telangiectasia mutated
B2MG: beta‐2 microglobulin
CDC6: cell division cycle 6
CDKIs: cyclin‐dependent kinase inhibitors
CHK2: checkpoint kinase 2
DAPI: 4′,6‐diamidino‐2‐phenylindole
DDR: DNA damage response
DEP1: density‐enhanced phosphatase‐1
DNA: deoxyribonucleic acid
DPP4: dipeptidyl peptidase‐4
EdU: 5‐ethynyl‐2′‐deoxyuridine
FFPE: formalin‐fixed and paraffin‐embedded
GCA: giant cell arteritis
H3K9me3: Histone H3 Lysine 9 Trimethylation
HMGA: high mobility group A
HMGB1: high mobility group box 1
HP1: heterochromatin protein 1
IF: immunofluorescence
IFN‐I: type I interferon
IHC: immunohistochemistry
IHF: immunohistofluorescence
MBS1: moebius syndrome 1
ML: machine learning
OIS: oncogene‐induced senescence
PCNA: proliferating cell nuclear antigen
PML Bodies: promyelocytic leukemia nuclear bodies
RAS: rat sarcoma viral oncogene homolog
RB: retinoblastoma protein
RNA‐ISH: RNA *in situ* hybridization
RNA‐Seq: ribonucleic acid sequencing
ROS: reactive oxygen species
RPS14: ribosomal protein S14
RS: replicative senescence
RT‐qPCR: reverse transcription quantitative PCR
SADS: senescence‐associated distension of satellites
SAHF: senescence‐associated heterochromatin foci
SASP: senescence‐associated secretory phenotype
SA‐β‐Gal: senescence‐associated‐β‐galactosidase
SBB Staining: Sudan Black B
scRNA‐seq: single‐cell RNA sequencing
SIPS: stress‐induced premature senescence
TAFs: telomere‐associated foci
TIFs: telomere dysfunction‐induced foci
TIS: therapy‐induced senescence
γH2AX: phosphorylated H2AX

## Cellular senescence: A glance at key concepts

Cellular senescence, first identified over 60 years ago by Hayflick and Moorhead [1], constitutes a stress‐response mechanism that is initiated when various intrinsic and extrinsic stressors jeopardize the cell's equilibrium [2, 3, 4]. Their pioneering work in human fibroblasts unveiled that cells do not divide indefinitely in cell culture; instead, they undergo a finite number of divisions and then progressively slow down, ultimately entering a nondividing state, termed senescence [3]. The threshold of these cellular divisions, known as the “Hayflick Limit,” was subsequently coined in Macfarlane Burnett's book Intrinsic Mutagenesis, published in 1974 [5]. Notably, the discovery of senescence emerged as a major turning point in cellular biology, challenging the prevailing dogma of cellular immortality, established by Alexis Carrel's earlier work [6]. Senescence was later mechanistically linked to telomere attrition, providing a biological basis for what is now known as Replicative Senescence (RS). Telomeres, regions of repetitive DNA sequences at the end of chromosomes, shorten with each replication passage. Upon reaching the “Hayflick Limit,” shortened telomeres are recognized as sites of DNA damage, triggering the DNA damage response (DDR) pathway and eventually leading to cell cycle arrest [7, 8]. As such, the propagation of “aged” cells is prevented [9, 10].

Over the past decades, it has become palpable that Hayflick and Moorehead's observations just uncovered the tip of the iceberg. Nowadays, it is well‐established that senescence can be triggered not only by telomere shortening but also by a variety of stressors independent of telomere length, collectively known as Stress‐Induced Premature Senescence (SIPS). Oncogene‐Induced Senescence (OIS), which results from the activation of oncogenes such as *RAS*, *BRAF*, *CDC6*, *AKT*, *CYCLIN* E, and *E2F1* [11, 12], is among the most notable types of SIPS. Additionally, SIPS can be the outcome of a wide array of factors and conditions ranging from DNA damage and oxidative stress to inflammation and even exposure to irradiation and pharmacological agents, with the latter resulting in a special type termed “Therapy‐Induced Senescence” (TIS) [3].

Despite the fact that cellular senescence is a hallmark of aging, it constitutes an entirely distinct phenomenon [13] as it can occur independently of age during an organism's lifespan [14, 15, 16]. It represents a highly complex and dynamic phenotype that largely depends on various factors, such as cell type, tissue context, species, and the nature of the inducing stimuli. This inherent complexity has led to the development of various methodologies, both interdependent and autonomous, for its effective identification and characterization across diverse biological contexts. Regardless of the setting, senescent cells exhibit four defining hallmarks: (i) a stable and generally irreversible cell‐cycle arrest, (ii) a senescence‐associated secretory phenotype (SASP), (iii) macromolecular damage, and (iv) altered metabolism [3].

Each hallmark is orchestrated through complex molecular events and acts dynamically to establish and maintain the senescent phenotype. First and foremost, cell cycle withdrawal results from the sequential activation of two key molecular pathways. In early phases, the p53/p21^WAF/Cip1^ axis induces growth arrest, which is subsequently maintained by the activation of the p16^INK4A^/RB pathway, effectively sustaining the senescent phenotype. The SASP secretome, in turn, consists of a variety of factors (cytokines, chemokines, growth factors, proteases, and other bioactive molecules) that function via both autocrine and paracrine signaling pathways [3, 17, 18, 19]. SASP composition varies depending on cell type, senescence trigger, and time point (early vs late), further denoting its dynamic nature. In early stages, SASP plays a beneficial role by attracting immune cells and therefore promoting the elimination of senescent cells. However, in later stages, SASP exerts detrimental effects by sustaining chronic low‐grade inflammation in tissues [20, 21, 22, 23]. Additionally, macromolecular damage in senescence is most commonly attributed to persistent DNA damage (genomic/telomeric DNA damage), with other common types being associated with protein and lipid oxidation [24, 25, 26]. Lastly, metabolic dysregulation further amplifies the senescent phenotype, including changes in glycolysis and autophagy, as well as mitochondrial and lysosomal metabolism and turnover. These metabolic shifts often lead to increased reactive oxygen species (ROS) production, which in turn fuels macromolecular damage, perpetuating a vicious cycle [27, 28].

Cellular senescence exerts a bimodal action [17, 19, 29, 30], acting transiently under physiological conditions. It effectively contributes to organogenesis during embryonic development and predominantly facilitates the immune‐mediated elimination of defective cells during injury and repair processes in adult tissues, thereby maintaining tissue homeostasis. In contrast, when senescent cells are not properly removed, their prolonged presence alters normal tissue architecture via late‐phase SASP, exacerbating the emergence of various age‐related pathologies, including cancer, cardiovascular, pulmonary, renal, hepatic, and neurodegenerative disorders [23, 31, 32, 33, 34, 35, 36, 37, 38]. Particularly in cancer, among the most frequent age‐related diseases, senescence has a dual role. In early, precancerous stages, OIS exerts a tumor‐suppressive role, effectively restraining the propagation of potentially cancerous cells. However, in advanced stages, it can promote malignancies, either through the SASP or, more importantly, via the “escape from senescence” phenomenon [18, 39, 40, 41]. According to the latter, senescent cells can, under specific circumstances, re‐acquire proliferative capacity, thus challenging the long‐held assumption that senescence represents an irreversible state. As such, senescent cells can act as sources of resistance to traditional cancer therapies and contribute to tumor recurrence. This underscores the need to revise anticancer strategies by incorporating senolytic approaches aimed at eradicating senescent cells into first‐line treatment regimens [18, 42].

As already mentioned, following its initial discovery in cell culture and its pivotal role in physiology, pathology, and aging, senescence has evolved into a cornerstone of aging and disease‐related research. In line with this, its defining characteristics have been gradually leveraged for the development of a variety of biomarkers and molecular signatures aimed at enhancing its identification in biological samples. These methodologies range from traditional wet‐lab techniques, comprising individual cellular traits and single molecular markers, to advanced computational and combinatorial approaches enabled by the era of big data, including machine learning (ML), multi‐omic applications, and foundational statistical approaches. While substantial progress has been achieved, no single method to date can universally and unequivocally detect senescent cells across tissues, species, and modalities without significant limitations.

Herein, we focus on past, current, and emerging detection methodologies, providing a critical evaluation of their advantages and limitations, effectively laying the groundwork for future improvements and advancements.

## Senescence biomarkers—From traditional to novel approaches

For many years, the assessment of cellular senescence relied on individual, empirically derived biomarkers, primarily reflecting phenotypic traits or biochemical/molecular features associated with pivotal cellular processes such as the cell cycle, cellular damage, altered gene expression, epigenetic modifications, metabolic shifts, chromatin remodeling, and a distinct secretory phenotype (Table 1). Such approaches, although widely used, proved ineffective and unreliable, given that the senescence phenotype is a context‐dependent process, exerting heterogeneity and a multifaceted profile [43]. Moreover, many of the markers proposed are not senescence‐specific and can be observed in various other cellular contexts and responses. As such, they are often burdened with false‐positive outcomes during senescence detection, leading to inaccurate conclusions, ultimately jeopardizing the validity of the investigations.

**Table 1:** Senescence markers short overview.

Cellular function	Markers	Detection method	References
Lysosomal β‐gal activity	β‐Gal	Histochemical staining (pH 6)	[43, 44, 84, 85, 86, 87, 88, 89]
Lysosomal accumulation	Lipofuscin accumulation	Autofluorescence microscopy, Histochemical Staining (SSB), GL13, GLF16, Confocal Microscopy	[3, 46, 47, 59, 61, 90, 91]
Nuclear body formation	PML Bodies	IF	[15, 92, 93, 94, 95, 96, 97]
Chromatin condensation	SAHF (H3K9me3, HP1, MacroH2A)	DAPI staining, Microscopy	[3, 98, 99, 100, 101, 102]
Telomeric DNA damage foci	TIFs/TAFs	IF	[60, 99, 103, 104]
Chromatin decompaction of pericentromeric satellites	SADS	FISH, Fluorescence Microscopy	[60, 105, 106, 107]
Lipid droplets	ALISE	Colorimetric, IF	[37, 60, 108]
Cell surface proteins	DEP1, B2MG and DPP4	FC, IF, WB, qRT‐PCR, IHF	[17, 109, 110, 111, 112]
CDKIs	p16^INK4a^, p21^CIP1^, p15^INK4B^, p27^KIP1^	IHC, IF, WB, RT‐qPCR, RNA‐ISH	[9, 13, 45, 79, 99, 113, 114, 115, 116, 117, 118, 119, 120, 121]
Cell proliferation marker	Ki‐67, PCNA	IHC, IF, EdU	[99, 122, 123]
Oxidative stress marker	4‐HNE	IHC, IF	[124, 125]
Nuclear envelope integrity	Lamin B1	IHC, IF	[126, 127, 128, 129, 130, 131, 132]
Resistance to apoptosis markers	BCL family members (Bcl‐2, Bcl‐w, or Bcl‐xL)	IHC, IF, ELISA, FC, WB, qRT‐PCR	[17, 87, 133, 134, 135, 136, 137]
Transcriptional regulation (Senescence/Escape)	DEC1 (BHLHE40)	qRT‐PCR, WB, IF, IHC	[18, 112, 138, 139]
Apoptosis‐resistance	DCR2 (TNFRSF10D)	FC, IHC, IF	[17, 140, 141, 142, 143]
DNA damage markers	ATM, 53BP1, γH2AX, MBS1, CHK2	IHC, IF, RNA‐ISH, WB	[99, 144, 145, 146, 147, 148, 149, 150, 151, 152]
Retrotransposable elements	IFN‐1	ELISA, IHC, IF, Multiplex Assays	[99, 153, 154]
DNA chaperone activity	HMGB1	IHC, IF	[60, 155, 156, 157, 158, 159, 160]
Chromatin modulators	HMGA proteins	qRT‐PCR, WB, IF, ChIP, IHC	[100, 157, 161, 162, 163]
Tumor suppressor	ARF	qRT‐PCR, WB, IF, IHC	[164, 165, 166]
Ribosomal proteins	The ribosomal protein S14 (RPS14/uS11)	ELISA, IHC, IF, Multiplex Assays	[167]
Interleukins	IL‐1 family (IL‐1, IL‐1b), IL‐6, IL‐7, IL‐8, IL‐13, IL‐15	ELISA, IHC, IF	[168, 169, 170, 171]
Chemokines	GRO (α, β, γ), MCP (2, 4), MIP (1α, 3α), HCC‐4, eotaxin (1, 3), TECK, ENA‐78, I‐309, I‐TAC	ELISA	[99, 168, 169]
Inflammatory molecules	TGFβ, GM‐CSF, G‐CSF, IFN‐γ, BLC, MIF	ELISA, IHC, IF	[168, 169, 172]
Proteolytic enzymes & inhibitors	MMPs (1, 3, 10, 12, 13, 14), TIMPs (1, 2), PAIs (1, 2), tPA, uPA, cathepsin B	ELISA, IHC, IF	[3, 168, 173]

Considering the above, it became apparent that no individual marker or method can universally and accurately detect senescent cells, effectively discriminating them from other non‐senescent states across tissues, species, and modalities. For instance, the most widely applied method in the senescence field, the Senescence‐Associated β‐Galactosidase (SA‐β‐Gal) assay, and related methodologies that detect enzymatic activity, are known to yield false‐positive and false‐negative results. In addition, these assays are not applicable in formalin‐fixed and paraffin‐embedded (FFPE) material. This limitation has rendered the identification of senescent cells *in vivo* unfeasible for many decades, highlighting the imperative need for new, more reliable markers and methods [3, 44, 45].

Toward this direction, our research provided conclusive evidence that lipofuscin accumulation is a hallmark of senescent cells [46, 47], expanding on prior indications linking lipofuscin with aging [48, 49, 50, 51]. Lipofuscin was initially observed over a century ago and is often described as the “dark matter” of the cell [18]; much like its cosmological counterpart, it is pervasive, accumulates over time, and the intricacies of its architecture remain to be explored. The term lipofuscin roughly translates in Latin to “dark lipid,” a description that only partially reflects its heterogeneous composition [52, 53]. This yellow‐brown autofluorescent pigment is primarily composed of oxidized proteins, lipids, and metals [54, 55]. Its accumulation is the most constant and common feature in senescence, mirroring macromolecular damage, deregulated metabolism, and cell cycle arrest, processes shared by all senescent cells [46, 56]. In some instances, translation deregulation mechanisms can also be involved in its aggregation [57]. This unique property has been exploited for the development of novel reagents (GL13, GLF16), which are Sudan Black‐B (SBB) [58, 59] analogues. These compounds strongly interact with lipofuscin, achieving accurate senescence detection in any type of biological material (GL13), including FFPE tissues [46, 47, 59], addressing previous limitations of the SA‐β‐Gal assay. Interestingly, GLF16, a hydrophilic, fluorophore‐conjugated SSB analogue, enabled the identification of live senescent cells for the first time, upon delivery via a nanocarrier and established *in vivo* senescence models. This approach also allowed the isolation of these cells, enabling in‐depth analysis at the genetic, epigenetic, and transcriptomic levels [46, 59]. These reagents detect lipofuscin, the common denominator of senescence, which can be used in conjunction with other biomarkers of senescence. Thus, they were promptly incorporated into a guideline, multimarker algorithm for accurate senescence detection [3, 60].

This development represented a turning point in the senescence field, surpassing the challenges and misleading outcomes frequently emerging from the use of single markers. According to this pipeline, lipofuscin detection should precede screening for senescence and should subsequently be followed by verification of the senescence phenotype, using biomarkers commonly associated with it (e.g., p21^WAF/Cip1^, p16^INK4A^, IL1b, IL‐6), as well as those that are typically absent in senescent cells (e.g., Ki67, Lamin B1). The application of the two aforementioned detection steps, according to Kohli and colleagues [61], in the frame of an algorithm allows for the specific assessment of cellular senescence in clinical material. Thus, by implementing this algorithm in various clinical specimens, we detected and proved the implication of senescent cells in various human pathological settings, such as cancer [17, 62, 63], COVID‐19 [64], and in giant cell arteritis (GCA) [65], opening new horizons for their treatment. Particularly, we have shown that increased senescence in Hodgkin and Reed Sternberg cells in Hodgkin Lymphomas is followed by adverse clinical outcomes [17]. In COVID‐19 lungs, alveolar type II (AT‐II) cells infected by SARS‐COV‐2 were demonstrated to exhibit cellular senescence and a pro‐inflammatory phenotype, putatively favoring the initiation of a lethal cytokine storm. These infected senescent cells were also shown to act as a fertile environment for SARS‐COV‐2 quasispecies generation [64]. Moreover, in GCA patients, high levels of senescent fibroblasts, macrophages, and endothelial cells were identified in their arteries compared to controls [65]. Importantly, apart from exploiting the SBB compounds for detection, they served as a backbone to generate a senolytic platform that selectively eliminates senescent cells *in vivo* (in established senescence animal models), without side effects [66].

The aforementioned algorithm introduced into the senescence field the concept of applying combinatorial markers/methods for precise senescence identification. In this context, advances in high‐throughput technologies and the availability of big data in recent years have led to the development and application of molecular signatures of senescence, with the purpose of better characterizing its complex phenotype, complementing and enhancing traditional approaches, or serving as the only option when biological material is unavailable. Additionally, these tools allow for retrospective assessment of senescence, revealing information that may otherwise remain hidden in various experimental and clinical settings, as discussed in the next section.

## Decoding senescence: A practical assessment of gene sets and resources

The identification of senescent cells across tissues and datasets, as previously mentioned, constitutes a topic of broad application and therefore scientific interest. Advances in high‐throughput technologies have enhanced the detection of cellular senescence, particularly through the development and application of molecular signatures that define this complex phenotype. When applied across diverse datasets, these signatures can effectively overcome the limitations of traditional senescence biomarkers. Additionally, they offer vital insights into cellular senescence while simultaneously enabling the retrospective detection of senescence states that might otherwise be overlooked across various pathologies.

Over the past years, numerous molecular signatures of senescence have been developed, each employing distinct methodologies and validation protocols. However, these signatures often differ to great extents in their defining characteristics, reflecting fundamental discrepancies in the data and contexts from which they originate. Upon deeper investigation, it becomes evident that these signatures are highly case‐specific, extracted from individually unique and fundamentally heterogeneous high‐throughput data, thus capturing only specific aspects of senescence in certain tissues, settings, or species. Consequently, a universally applicable signature, capable of reliably detecting senescence across modalities, is still lacking.

Most of the currently available molecular signatures have been developed through the utilization of omic‐based technologies and datasets, such as single‐cell or bulk RNA sequencing, proteomics, metabolomics, and epigenomics. However, only a few of them have undergone thorough experimental validation, resulting in ambiguous evidence regarding their consistency with these methodologies. Thus, this lack of cross‐validation raises concerns regarding the extent to which *in silico* findings align with their experimental counterparts. These observations highlight the need for a meta‐review of the already established senescence molecular signatures in order to adequately compare and characterize their advantages and disadvantages.

In this review, we performed an extensive curation of the current literature, compiling a substantial body of data to critically assess the prevailing methodologies for senescence identification. Our analysis and findings considered three distinct categories: Senescence molecular signatures, Databases, and Computational Models, with a focus on molecular signatures. Briefly mentioned below are the tools and methodologies currently available for the *in silico* identification of senescent cells.

1. Senescence molecular signatures: Gene sets characterizing senescence (Table 1).
2. Databases: Publicly available repositories‐resources regarding senescence, with the most validated one's being: CellAge [67], SeneQuest [3], CsGene [68], GenAge [69], HAGR [70], SenOmic [71], HCSGD [72], SenNet [73].
3. Computational models: Statistical and computational approaches that aim to identify senescent cells in different environments. Namely: DeepScence [74], hUSI [75], SenCID [76], SenPred [77], D‐MAINS [78], Duran et al [79].

**Fig. 1:**
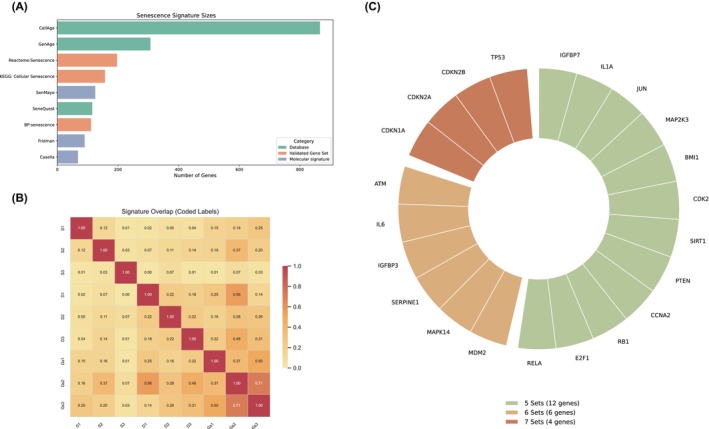
Comparative analysis of molecular gene lists defining senescence. (A) A quantitative comparison of all gene lists highlighting substantial variability in their sizes. The X‐axis presents the number of genes derived from their respective publications, whereas the Y‐axis depicts the individual senescence gene resources. (B) Overlap analysis revealing limited intersection among gene sets. Abbreviated names are set as follows: “SenMayo” as S1, “Fridman” as S2, “Casella” as S3, “BP: senescence” as D1, “Reactome: Senescence” as D2, “KEGG: Cellular Senescence” as D3, “GenAge” as Gs1, “CellAge” as Gs2, and “SeneQuest” as Gs3. (C) Pairwise comparative analysis revealing a core gene signature shared among most gene lists. Wedge colors indicate how many senescence gene resources contain each gene.

As an initial step, a small‐scale comparative analysis of molecular signatures, databases, and curated/pathway‐based gene sets was performed to ascertain some initial quantitative characteristics and to derive a core gene signature shared across them (Fig. 1C). To align with the objectives of this review, the analysis was conducted using the top three most validated candidates from each of the aforementioned categories. SeneQuest, a literature‐based evidence database of genes related to senescence, which is publicly available [3], was filtered to only retain genes with a combined annotation score (published evidence of gene upregulation or downregulation) of 15 or higher, thereby excluding poorly reviewed genes that are unlikely to play an active role in the senescent phenotype.

As illustrated in Fig. 1A, there is considerable quantitative heterogeneity among the examined gene lists, with most displaying large discrepancies in the number of genes identified. This variability likely reflects the nature of their technical development (different methodologies, tissues, and other factors) as well as their lack of specificity, including genes not directly related to senescence. Subsequently, an overlap analysis of genes between all categories was carried out (Fig. 1B), revealing limited overlap, suggesting that most of them capture only specific facets of the senescence phenotype rather than its full spectrum. For practical purposes, the signatures and datasets were renamed as follows in Fig. 1B: “SenMayo” as S1, “Fridman” as S2, “Casella” as S3, “BP: senescence” as D1, “Reactome: Senescence” as D2, “KEGG: Cellular Senescence” as D3, “GenAge” as Gs1, “CellAge” as Gs2, and “SeneQuest” as Gs3.

As a result of this comparative analysis, a core gene set consisting of 22 genes emerged (Fig. 1C). These genes are present in at least five of the aforementioned gene lists, highlighting their importance for the establishment of senescence. Specifically:

1. Appearing in 7 sets: *CDKN1A*, *CDKN2A*, *CDKN2B*, *TP53*
2. Appearing in 6 sets: *IGFBP3*, *IL6*, *SERPINE1*, *MDM2*, *MAPK14*, *ATM*
3. Appearing in 5 sets: *IGFBP7*, *IL1A*, *JUN*, *MAP2K3*, *BMI1*, *CDK2*, *SIRT1*, *PTEN*, *E2F1*, *RB1*, *CCNA2*, *RELA*

As displayed, cellular senescence appears to be regulated by a network of tumor suppressor genes. Among the most consistently validated genes across these signatures are *CDKN1A*, *CDKN2A*, *CDKN2B*, and *TP53*. In response to cellular stress, *TP53* is activated and in turn induces *CDKN1A*, which imposes cell cycle arrest at the G1/S checkpoint by inhibiting *CDK2* activity [80, 81]. In parallel, *CDKN2A* and *CDKN2B* function through the *RB* pathway, reinforcing cell cycle arrest independently of *p53* through *CDK4/6* inhibition and *RB* activation [80]. These genes, which besides *TP53* constitute essential biomarkers of senescence as displayed in Table 1, further support the validity of our findings.

Following this small‐scale analysis, we developed a comprehensive comparison table (Table 2), effectively comparing senescence molecular signatures for various predefined criteria. These criteria, namely: (i) sensitivity, (ii) specificity, (iii) cellular diversity, (iv) tissue coverage, (v) cross‐species applicability, (vi) and high‐throughput (HT) data validation, even though not exhaustive, represent the main features that define these signatures and therefore can be used as a preliminary tool to evaluate them. Notably, the cross‐species metric constitutes a crucial requirement for the applicable effectiveness of a signature, given its impact and implication in the rest of the evaluation criteria, allowing for its broader application and validation. As is evident, no signature unifies all the aforementioned metrics.

**Table 2:** Senescence Molecular Signatures Comparison.

Signature name	Sensitivity	Specificity	Diverse cellular origin	Detection across tissues	Cross‐species detection	HT data validation	References
SenMayo	✓	✗	✓	✓	✓	✓	[174]
Fridman	✓	✓	✓	✓	✗	✗	[175]
Purcell	✓	✓	✗	✓	✓	✗	[176]
EndoSEN	✓	✓	✗	✗	✓	✓	[177]
Casella	✓	✓	✓	✓	✗	✗	[178]
Hernadez‐Segura	✓	✓	✗	✓	✓	✓	[120]
De Cocco	✗	✗	✓	✓	✗	✗	[153]
SenSig	✓	✓	✗	✓	✓	✓	[179]
SenSkin	✓	✓	✗	✗	✗	✓	[180]
D.Saul	✓	✗	✓	✓	✗	✓	[181]
S.Olascoaga	✗	✗	✗	✗	✗	✓	[182]

All six of the aforementioned criteria, originating from different scientific fields, such as mathematics or systems biology, provide solely valuable information. However, when considered in unison, they constitute a multiparametric framework covering all the basics a molecular signature must exhibit in order to be deemed reliable. Before diving into the comparison itself, it is important that we briefly introduce and define each of these criteria. Starting off with the mathematically derived criteria, namely sensitivity and specificity. These constitute statistical measures commonly used in life sciences to either measure the proportion of true positives correctly identified (sensitivity) or to measure the proportion of true negatives correctly identified (specificity) [82]. In our case, these two metrics effectively represent how well a signature is in detecting senescent cells even at low abundance or how well it avoids characterizing non‐senescent cells (e.g., quiescent cells) as senescent ones, respectively. The combination of these two enhances the reliability and interpretability of *in silico* senescence assessment approaches.

Additionally, besides evaluating signatures on their identification accuracy, it should be taken into account how well this accuracy translates in different contexts. To this end, cellular diversity and tissue coverage effectively ascertain that, at the very minimum, a signature has wide enough coverage in order to be used broadly and without contextual constraints. On this basis, signatures must be derived from a wide array of settings (tissues, conditions, inducers, and combinations thereof) so that they are able to efficiently identify a phenomenon (in our case, senescence) universally. Ultimately, these two metrics present a cause‐and‐effect relationship that defines a signature's range of applicability. Building on the aforementioned observations, a signature must be cross‐species applicable. This is a crucial element of its arsenal, as it enables integration and validation across multiple studies and parameters and, in turn, facilitates its application in high‐throughput datasets, thereby covering all six criteria. High‐throughput dataset validation places the final piece into the framework due to the reliability of its findings. In line with the current state of big data, consistent high‐throughput data validation is one of, if not the most, accurate ways to validate the applicability of any molecular model (e.g., a signature) in a vast contextual field, therefore ensuring reliability in a statistically acceptable manner.

Based on the above, a major limitation of several signatures lies in their narrow applicability and validation across only certain tissues or cell types. This usually originates from the inherent complexity of senescence and consequently the computational and biological difficulties arising during these processes. In that notion, Purcell, EndoSEN, SenSkin, and S. Olascoaga were derived exclusively from fibroblasts with Li‐Fraumeni syndrome, skin fibroblasts, endothelial cells, and prostate epithelial cells, respectively. This fact renders them highly specialized for tissues similar to those they were derived from, severely restricting their applicability in other biological contexts. In contrast, SenMayo, FridMan, D. Saul, and Casella are based on data from multiple cell types and tissues, thus effectively making them applicable to a wider context. This metric constitutes one of the most important features when evaluating a molecular signature and should therefore always be assessed both during its creation and during subsequent validations.

Delving further into the publications of all the signatures in order to assess the cross‐species validation metric, signatures such as SenMayo, Purcell, EndoSEN, Hernandez‐Segura, and SenSig have been directly evaluated for their applicability in both human and murine settings. They have demonstrated valuable results in murine models, although EndoSEN has undergone testing in endothelial cells and therefore has a narrower significance. By contrast, signatures such as Fridman, Casella, De Cocco, SenSkin, D. Saul, and S. Olascoaga concern exclusively human datasets, with their cross‐species validation remaining unclear. The direct and in‐depth cross‐species validation remains a key limitation for most of these tools.

For example, the De Cocco signature, based on Type I interferon (IFN‐I) signaling, captures an important dimension of late SASP, with its specificity, however, limited due to the fact that IFN‐I signaling is not unique to senescence. Similarly, the S. Olascoaga signature, designed to detect oxidative stress‐induced senescence, may be less effective against other senescence types. Although D. Saul is able to assess senescence in multiple tissues, it seems to be less effective in certain cell populations. The aforementioned examples underscore the need for signatures to be responsive to multiple senescence triggers but, in the same context, remain specific to the senescence phenotype and not get confounded by neighboring biological processes.

When considering applicability across senescence‐inducing stimuli, only the SenMayo, Purcell, EndoSen, Casella, and Hernandez‐Sengura signatures appear adequate across different senescence types, including RS, OIS, and TIS. Of note, SenMayo, D. Saul, Hernandez‐Segura, SenSig, and SenSkin have each been assessed in high‐throughput datasets, including bulk and single‐cell data.

The vast majority of the aforementioned molecular signatures of senescence remain insufficiently validated across multiple high‐throughput modalities and biological contexts. Specifically, they lack systematic cotesting across diverse tissue types, inducing stimuli or high‐throughput techniques such as bulk RNA sequencing or single‐cell RNA sequencing (scRNA‐seq). Even though they still offer great practical detection value in narrow contexts, this drawback severely hinders their reliability and universality. In contrast, systematically derived signatures (e.g., SenMayo) allow validation in a wide variety of data sources, effectively overcoming the aforementioned limitations.

Complementing the above, and prominent even among the most promising signatures including SenMayo, SenSig, D. Saul, SenSkin, Hernandez‐Segura, De Cocco, and S. Olascoaga, is the lack of gene expression directionality. Without providing adequate annotation on whether the genes are up‐ or downregulated in senescence, there is a great loss of practical information, especially useful during the interpretation of their results. Usually, this issue is mildly overcome in computational settings when genes are assigned some sort of metric, a ranking score, weights, or enrichment scores, and therefore showing its practical importance in said context.

Lastly, while Fridman and Casella signatures are considered among the most promising ones, mainly due to their inclusion of biomarkers and their multitype senescence validation, they remain untested in scRNA‐seq data and lack cross‐species validation. These limitations, even though balanced by their advantages, represent critical weaknesses, especially during the era of single‐cell omics and in the current state of senescence research.

## Concluding remarks and future perspectives

Despite a growing scientific interest and the increasing number of senescence signatures, no existing individual tool is able to fully capture the senescent phenotype across diverse biological systems and omics platforms. Most currently used signatures are narrowly focused on specific cell types or have very limited cross‐modality validation, effectively reducing their practical applicability and effectiveness. While some signatures, such as SenMayo and Fridman et al., demonstrate great potential, gaps still exist in their interpretability and cross‐context applicability. Additionally, the absence of a standardized benchmarking framework for adequate evaluation renders their practical assessment inconsistent, hindering their selection for application in clinical or experimental settings.

Based on the above, there is an imperative need for the development of a signature that will be able to address the aforementioned limitations and gaps in the field, effectively integrating multi‐omic data (transcriptomic, proteomic, and epigenomic), spanning diverse cellular states, displaying gene directionality, and properly validated across species, tissues, and perturbation models [83]. This would effectively enable the discovery of previously overlooked aspects of the senescence phenotype through the reanalysis of data curated using older signatures, while also establishing the background for facilitating further improvements.

## Conflict of interest

The authors declare no conflict of interest.

## Author contributions

OAN, PP, and SV performed the literature search. PP conducted the literature‐based comparison of senescence signatures. Data analysis was carried out by OAN. OAN, PP, RP, and KE wrote the manuscript. GT and SV prepared the figures. VGG supervised the current work. All authors reviewed and approved the final manuscript.
